# Impact of demographic and psychosocial factors on hysterosalpingography pain and discomfort

**DOI:** 10.4102/phcfm.v1i1.40

**Published:** 2009-06-30

**Authors:** Anthony C. Ugwu, Augustine O. Imo, Okey F. Erondu

**Affiliations:** 1 Department of Medical Radiography and Radiological Sciences, Nnamdi Azikiwe University, Nigeria; 2 Department of Radiology, Ebonyi State University Teaching Hospital, Nigeria; 3 Department of Physics (Medical Physics), Rivers State University of Science and Technology, Nigeria

**Keywords:** hysterosalpingography, pain, discomfort, radiographic delineation, infertility

## Abstract

**Background:**

Hysterosalpingography (HSG) is an important diagnostic procedure in the investigation of infertility. It is the radiographic delineation of uterine and tubal cavities and is part of the diagnostic evaluation of conjugal infertility.^1^ This diagnostic procedure is associated with high levels of anxiety, pain and stress from various causes. This study was designed to investigate the impact of demographic and psychosocial factors on HSG pain and discomfort.

**Method:**

One hundred hysterosalpingography referrals were recruited for this study. Verbal detector scales were used to assess pain perception, Likert scales were used to assess the psychosocial variables, while visual analogue scales were used to assess discomfort. Pearson's correlations were conducted. Tests were two-tailed, with p < 0.05 indicating statistical significance.

**Results:**

Some of the patients (34%) indicated that the administration of analgesics prior to the procedure reduced the pain and discomfort associated with the procedure. Mean ± standard deviation of pain and discomfort were 2.82 ± 0.77 and 6.36 ± 2.19 respectively. Age correlated significantly with pain perception (r = -0.22, P < 0.05), while pain correlated significantly with perception of discomfort (r = -0.46, P < 0.05).

**Conclusion:**

Age significantly correlated with pain. This is a factor that could be harnessed for clinical use.

## INTRODUCTION

Hysterosalpingography (HSG) is an important diagnostic procedure in the investigation of infertility. It is the radiographic delineation of uterine and tubal cavities and is part of the diagnostic evaluation of conjugal infertility.^1^ This diagnostic procedure is associated with high levels of anxiety, pain and stress from various causes.^2, 3^ Up to 72% of women complain of discomfort caused by HSG.^4^ This can have a negative impact on the patient's ability to fully cooperate with the procedure, as well as on her willingness to undergo a repeat of the procedure or even take other diagnostic tests.

The difference between the notions of ‘pain’ and ‘discomfort’ has remained unclear when only one or the other has between measured,^5, 6, 7^ or both have been combined on the same scale.^8, 9^ The medical literature considers comfort more in its absence, for instance in the assessment of discomfort in mammography,^10, 11^ with discomfort regarded as the most painless version of pain. However, discomfort has also been defined in the medical world as a subjective, unpleasant feeling that the patient does not interpret as pain.^12^ Comfort is considered a multidimensional construct covering the physical, social, psychospiritual and environmental dimensions.^13^

The enhancement of comfort is one of the primary goals of radiographers, nurses and radiologists carrying out hysterosalpingography (uterosalpingography). It is a common practice to prepare patients psychologically before the procedure. A good knowledge of the impact of demographic and psychosocial factors on HSG pain and discomfort would act as a good guide to radiographers, radiology nurses and radiologists. This study was designed to investigate the impact of demographic and psychosocial factors on HSG pain and discomfort. To the best of our knowledge, this work has never been done before.

## METHOD

This study evaluated a convenience sample of one hundred (100) patients referred for HSG for the evaluation of infertility in two centres in southeast Nigeria from June 2007 to March 2008. Ethics approval and the patients’ consent were obtained. The traditional technique performed with a metal cannula was adopted for all the patients included in this study. The procedure was always performed in accordance with the ten-day rule. Exclusion factors included the use of any medication that increases or decreases neurological pain thresholds, and any disease process associated with increasing or decreasing the pain threshold (such as constipation, chronic pelvic pain, lower back pain or diabetes). The patients underwent HSG via the metal cannula technique, with no anaesthesia being used. The technique consists of bimanual examination, followed by placement of the speculum, vaginal antisepsis, gripping of the anterior lip of the cervix with forceps, and coaptation of the metal cannula into the external cervical orifice for the injection of a hydro-soluble iodinated contrast medium (20 ml of 76% urografin). The contrast agent was introduced and radiographs were obtained. Each patient completed a questionnaire at the end of the procedure.

The questionnaire contained three sections, A, B and C. Part A was designed to collect demographic information, e.g. age, academic qualification and occupation. Lower academic qualifications (secondary, primary school and teachers grade 11 certificates) were coded 1, while higher academic qualifications (degrees, higher and lower diplomas, and the Nigeria Certificate in Education) were coded 2. Students and private sector-based patients were coded 3 (occupation), while public servants were coded 4. Part B of the questionnaire adopted ordinal scales. A four-point Likert format was used to measure psychosocial variables. The variables included the patients’ perceptions of the explanations of the procedure prior to the investigation, their fear of contagion, satisfaction with the procedure, the privacy provided, and time spent on the procedure. Strongly disagree was rated 1, disagree was rated 2, agree was rated 3 and strongly agree was rated 4. A four-point verbal descriptor scale (VDS) was used to assess the patients’ perceptions of pain during the procedure. Not painful at all was rated 1, painful was rated 2, very painful was rated 3 and extremely painful was rated 4. A ten-point visual analogue scale (VAS) of 1 to 10 was designed and used to assess the patients’ perceptions of discomfort during the procedure. A high score on the scale indicated a high level of discomfort and a score of 1 denoted no discomfort at all. Part C was an open-ended question that sought suggestions from the patients on how the pain and discomfort associated with the procedure could be reduced.

For the statistical analysis, the test for normality was carried out using the three-sigma rule. The descriptive analysis involved the determination of the mean, standard deviation (SD), mode, median and skewness. In the inferential statistical analysis, Pearson's correlation was used to obtain the relationship between occupation, educational qualification and psychosocial variables. The content of comments made in part C of the questionnaire was analysed. The comments were subjected to theme analysis by grouping the themes to establish major areas of agreement. SPSS 7.5 software was used for analysis. All tests were performed with a 5% significant level.

## RESULTS

A total of one hundred (100) questionnaires was distributed and collected. The ages of the patients enrolled in this study ranged from 23 to 46, with a median age of 32. The mean age ± SD was 32.57 ± 6.05, while the modal age was 30. 52 patients (52%) had lower academic qualifications, while 48 (48%) had higher educational qualifications. 52 of the patients (52%) were students or based in the private sector, while 48 (48%) were public servants. In this study, pain perception correlated negatively and significantly with discomfort occasioned by HSG (r = -0.46, p < 0.05). Table 1 shows the descriptive statistics of some of the measured variables.

**TABLE 1:** Descriptive statistics of some measured variables

VARIABLE	MEAN ± SD	MEDIAN	MODE	SKEWNESS
Perception of explanation about the procedure	2.939 = 0.87	3.00	3.00	-0.810
No fear of contagion	2.75 ± 0.85	3.00	3.00	-0.313
Satisfaction rating	3.28 ± 0.53	3.00	3.00	-0.262
Perception of privacy	3.10 ± 0.75	3.00	3.00	-0.463
Time taken during examination	3.04 ± 0.74	3.00	3.00	-0.526
Perception of discomfort	6.36 ± 2.19	6.50	5.00	-0.402
Perception of pain	2.82 ± 0.77	3.00	3.00	-0.187

Table 2 shows the Pearson's correlation coefficients and their p values between educational qualification and occupation with some psychological variables. Positive correlations are in favour of variables with higher codes. Table 3 shows the Pearson's correlation coefficients and their p values between HSG pain and discomfort with some measured variables.

**TABLE 2:** Pearson's correlation coefficients (r) and their p-values between educational qualifications (codes 1 & 2) and occupation (codes 3 & 4) with psychosocial factors

	QUALIFICATIONS		OCCUPATION	
		
VARIABLES	r	P-VALUE	r	P-VALUE
Perception of explanation of the procedure	0.27	0.007 (S)	-0.061	0.545 (NS)
No fear of contagion	-0.12	0.238 (NS)	0.119	0.238 (NS)
Satisfaction rating	-0.10	0.339 (NS)	-0.017	0.870 (NS)
Perception of privacy	0.03	0.749 (NS)	-0.076	0.455 (NS)
Time taken for examination	0.25	0.013 (S)	0.250	0.013 (S)
Perception of discomfort	0.01	0.948 (NS)	-0.04	0.698 (NS)
Perception of pain	0.043	0.67 (NS)	0.088	0.385 (NS)

NS = Not significant, S = significant, r = correlation coefficient, P = statistical level of significance

**TABLE 3:** Pearson's correlation coefficients and their p-values between HSG pain and discomfort with some measured variables

VARIABLE	PAIN		DISCOMFORT	
		
	r	P-VALUE	r	P-VALUE
Age	-0.22	0.03 (S)	-0.174	0.083 (NS)
Perception of explanation of the procedure	-0.11	0.28 (NS)	0.06	0.546 (NS)
No fear of contagion	-0.085	0.40 (NS)	0.00	1.00 (NS)
Satisfaction rating	-0.12	0.23 (NS)	0.077	0.45 (NS)
Perception of privacy	-0.12	0.07 (NS)	-0.12	0.254 (NS)
Time (long) spent during the procedure	-0.04	0.68 (NS)	0.07	0.514 (NS)

Content analysis of part C (comments made by the patients) indicated that 34 patients (34%) believed that the administration of pain-relieving drugs (analgesics) would reduce the pain and discomfort occasioned by this procedure. One patient (1%) believed that prayer would solve the problem, while two patients (2%) suggested that friendliness from the workers would ameliorate the pain and discomfort associated with the procedure. Three patients (3%) suggested that professionals should do rigorous research to find out how best the pain and discomfort associated with HSG could be controlled, while 60 patients (60%) made no comments at all.

## DISCUSSION

Hysterosalpingography remains one of the first steps in the evaluation of a woman for infertility. Most studies have shown that it is more painful than sonohysterosalpingography or outpatient hysteroscopy,^14, 15^ which are examinations used for the same purpose. The authors investigated the impact of demographic and psychosocial factors on pain and discomfort experienced during HSG.

The study has shown that the mean age ± SD of the women in this study was 32.57 ± 6.05, the median age was 32 and the modal age was 30. These values are in agreement with those in a previous study by Agwu and Okoye,^16^ which reported that women between 25 and 34 years of age constituted the highest number of referrals for hysterosalpingography. Varpular obtained a VAS value of 5.2 for pain experienced during HSG with the use of metal cannula.^17^ On a ten-point scale, this value translates to 2.08 on the four point scale (VDS) used in this study. This value (2.08) is in agreement with the pain perception reported in the present study, and falls within the range of the mean ± SD value of pain perception reported in this study. Time spent refers to the patients’ perception of a long time. Patients with higher codes (higher qualification and public servants) have a greater tendency to believe that they have spent a lot of time, as shown by the significant relationship between qualification and perception of time spent.

None of the psychosocial variables correlated significantly with the perception of pain or discomfort. Educational qualification and occupation did not correlate significantly with pain and discomfort. A higher skewness (-0.402) value for discomfort than for pain indicates higher subjectivity and variability in the assessment of discomfort than of pain. Age was the only demographic factor that correlated significantly with pain (r = -0.22, P < 0.05). This implies that younger patients are more sensitive to pain and should be treated accordingly. It is therefore suggested that there should be an emphasis on pain management in the case of patients younger than the median age of 32 years.

The modern paradigm of pain management has moved from the biomedical to the broader biopsychosocial approach, where pain mechanisms integrate input from sensory, emotional and cognitive systems.^18, 19, 20^ The current definition of pain, as proposed by the International Association for the Study of Pain (IASP), reads: ‘pain is an unpleasant sensory and emotional experience associated with actual or potential tissue damage, or described in terms of such damage.’^21^ This definition identifies the complex and multidimensional experience of pain, where the patients’ physical, cognitive, emotional and behavioural characteristics mediate the pain experience.^22^ This multidimensional experience of pain is not in agreement with the present study, as all the psychosocial variables did not significantly correlate with pain. This difference could be the distinction between chronic and acute pain, as Katz^22^ considered the multidimensional nature of chronic pain.

Some of the issues we were unable to examine in this study invite further research. One such question is the existence or otherwise of pain anticipation and the source of such anticipation: media, friends, websites or healthcare professionals. Another is the impact of the attitude of the staff towards discomfort and pain experienced during HSG, patients’ methods of coping with pain and the role of coping strategies. Rigorous research in this area, as suggested by some of the patients, and an improvement in the staff attitude towards the patients are also recommended. Another limitation in this study was that the patients were not given analgesics prior to the study in order to reduce pain as an aspect of patient care concern. It is hereby suggested this ethical and patient care aspect should be taken into account in future studies. This study has established that age negatively and significantly correlates with HSG pain, which is a factor that could be used in the clinical environment in relation to reassurance and patient care.
